# *Auxenochlorella protothecoides* and *Prototheca wickerhamii* plastid genome sequences give insight into the origins of non-photosynthetic algae

**DOI:** 10.1038/srep14465

**Published:** 2015-09-25

**Authors:** Dong Yan, Yun Wang, Tatsuya Murakami, Yue Shen, Jianhui Gong, Huifeng Jiang, David R. Smith, Jean-Francois Pombert, Junbiao Dai, Qingyu Wu

**Affiliations:** 1 MOE Key Laboratory of Bioinformatics and Center for Synthetic and System Biology, Tsinghua University, Beijing 100084, China; 2 BGI-Shenzhen, Shenzhen 518083, China; 3 Key Laboratory of Systems Microbial Biotechnology, Tianjin Institute of Industrial Biotechnology, Chinese Academy of Sciences, Tianjing 300308, China; 4 Department of Biology, University of Western Ontario, London, Ontario, N6A 5B7, Canada; 5 College of Science, Illinois Institute of Technology, Chicago, IL 60616, USA

## Abstract

The forfeiting of photosynthetic capabilities has occurred independently many times throughout eukaryotic evolution. But almost all non-photosynthetic plants and algae still retain a colorless plastid and an associated genome, which performs fundamental processes apart from photosynthesis. Unfortunately, little is known about the forces leading to photosynthetic loss; this is largely because there is a lack of data from transitional species. Here, we compare the plastid genomes of two “transitional” green algae: the photosynthetic, mixotrophic *Auxenochlorella protothecoides* and the non-photosynthetic, obligate heterotroph *Prototheca wickerhamii*. Remarkably, the plastid genome of *A. protothecoides* is only slightly larger than that of *P. wickerhamii*, making it among the smallest plastid genomes yet observed from photosynthetic green algae. Even more surprising, both algae have almost identical plastid genomic architectures and gene compositions (with the exception of genes involved in photosynthesis), implying that they are closely related. This close relationship was further supported by phylogenetic and substitution rate analyses, which suggest that the lineages giving rise to *A. protothecoides* and *P. wickerhamii* diverged from one another around six million years ago.

There is a diversity of feeding strategies across the tree of life. Photoautotrophs, for instance, produce organic materials through photosynthesis and, thus, do not require exogenous organic matter. Heterotrophs, alternatively, survive on organic components from the environment. In eukaryotes, photosynthesis occurs in the chloroplast, which evolved circa 1.5 billion years ago through the endosymbiosis of a cyanobacterium by a unicellular, heterotrophic protist^1^,^2^. Despite the obvious benefits of photosynthesis, many eukaryotes have forfeited their photosynthetic capabilities, including various parasitic land plants and heterotrophic algae^3^,^4^,^5^. With few exceptions^6^, non-photosynthetic plants and algae still contain a colorless chloroplast (plastid) and a highly reduced plastid genome, both of which continue to carry out essential processes, apart from photosynthesis^3^,^5^,^7^,^8^,^9^,^10^. Among the leading models for understanding the evolutionary loss of photosynthesis are green algae^11^, including the colorless genera *Prototheca*, *Helicosporidium*, *Polytoma*, and *Polytomella*.

Currently, the only complete plastid genome sequence available from non-photosynthetic green algae is that of the trebouxiophyte *Helicosporidium* sp. ATCC 50920, which is a parasite of various invertebrates^12^. The *Helicosporidium* plastid DNA (ptDNA) is highly reduced (<40 kb), having lost various genes related to photosynthesis, and is similar in structure and content to the plastid genomes of apicomplexan parasites, such as *Plasmodium falciparum*^8^. The closest known relatives of *Helicosporidium* are from the non-photosynthetic trebouxiophyte genus *Prototheca*^13^, which is comprised of ubiquitous opportunistic animal pathogens, some of which can infect humans. Partial ptDNA sequence data from *Prototheca wickerhamii*^14^ suggest that its plastid genome is in a “transitional stage” between *Helicosporidium* and various photosynthetic trebouxiophytes. The closest known photosynthetic relative of *Prototheca* species is *Auxenochlorella protothecoides*^15^,^16^,^17^,^18^, a free-living green alga that can use sugars as carbon sources for heterotrophic growth^19^,^20^. When *A. protothecoides* cells are switched to heterotrophic conditions, their plastids can degenerate, resulting in the suppression and eventual elimination of photosynthesis^21^,^22^. Remarkably, this process is reversible, depending on the conditions, and suggests that *A. protothecoides* could provide insights into the loss of photosynthesis.

Here, in the hopes of better understanding the shift from a photoautotrophic to heterotrophic lifestyle, we report and compare the plastid genome sequences of *A. protothecoides* and *P. wickerhamii*. Both genomes show a surprising amount of similarities, including severe ptDNA contraction and similar gene orders and gene contents, photosynthesis-related genes notwithstanding. Our phylogenetic inferences and other genomic analyses confirmed that *A. protothecoides* and *P. wickerhamii* are indeed closely related, with a recent divergence time of about six million years. Together, our results provide interesting clues about the loss of photosynthesis and the evolution of obligate heterotrophy within green algae.

## Results

### The *A. protothecoides* and *P. wickerhamii* plastid genomes are paragons of compactness

**Figure 1:**
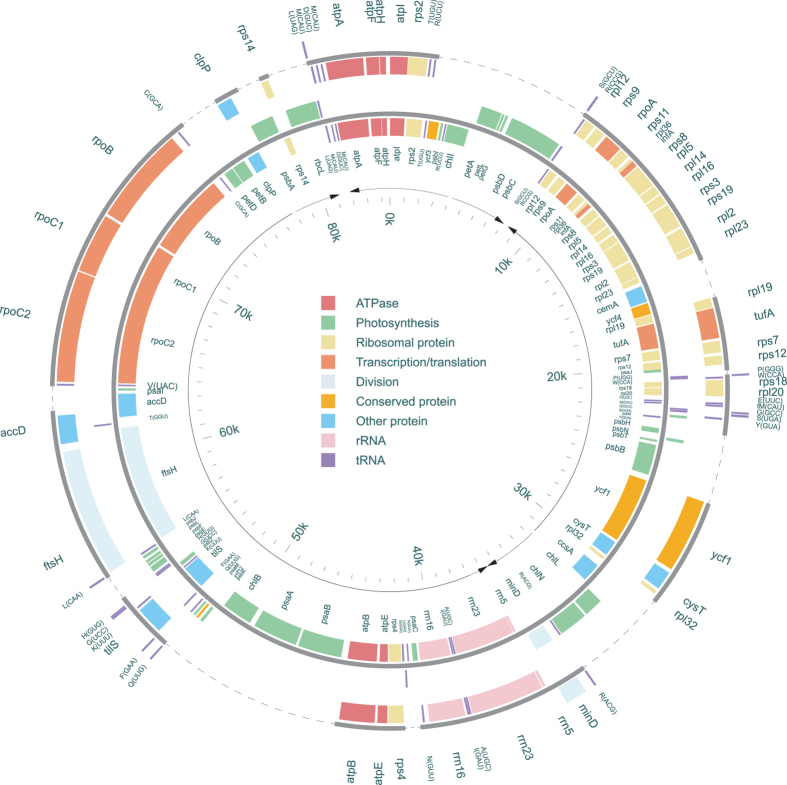
Gene maps of *A. protothecoides* and *P. wickerhamii* plastid genomes. The two concentric maps represent the ptDNAs of *A. protothecoides* (inner circle) and *P. wickerhamii* (outer circle), respectively. Genes (filled boxes) are color-coded into 9 groups according to their biological functions. Genes on the outside of each map are transcribed in a clockwise direction, whereas those on the inside of each map are transcribed counterclockwise (The direction of transcription is also pointed out by the black arrows). The tRNA genes are indicated by the one-letter amino acid code followed by the anticodon in parentheses. The dashed lines indicate regions absent from the *P. wickerhamii* genome.

The *A. protothecoides* plastid genome is an 84.58 kb, AT-rich (69.2%), circular-mapping molecule (Fig. 1; Supplementary Table S1). It has a compact architecture (24.57% non-coding), with no inverted repeats or introns. The *P. wickerhamii* ptDNA architecture mirrors that of *A. protothecoides*: it is small (55.64 kb), circular, AT-rich (68.8%), and compact (28.8% non-coding) (Fig. 1; Supplementary Table S2). These two genomes are among the smallest and most reduced ptDNAs observed from the Trebouxiophyceae (Table 1) and green algae as a whole (Supplementary Table S3). The genomic compaction of the *A. protothecoides* and *P. wickerhamii* ptDNAs largely results from being no introns and relatively little intergenic DNA (Table 1). Moreover, genes essential for photosynthesis have been lost in *P. wickerhamii* (discussed below). Further contributing to the ptDNA streamlining in *A. protothecoides* and *P. wickerhamii* is the lack of plastid inverted repeat elements. The absence of these elements, however, is a reoccurring theme throughout the Trebouxiophyceae (Table 1).

The types of plastid genome reduction observed in *A. protothecoides* and *P. wickerhamii* are not uncommon for green algae, especially non-photosynthetic species. In fact *Helicosporidium* sp. has one of the smallest ptDNAs ever observed^8^. Although only about 2/3 the size of that of *A. protothecoides*, the *P. wickerhamii* ptDNA is still larger and more expanded than that of *Helicosporidium* sp. (Reference^8^ and Table 1).

### *A. protothecoides* and *P. wickerhamii* have similar plastid gene contents

**Figure 2:**
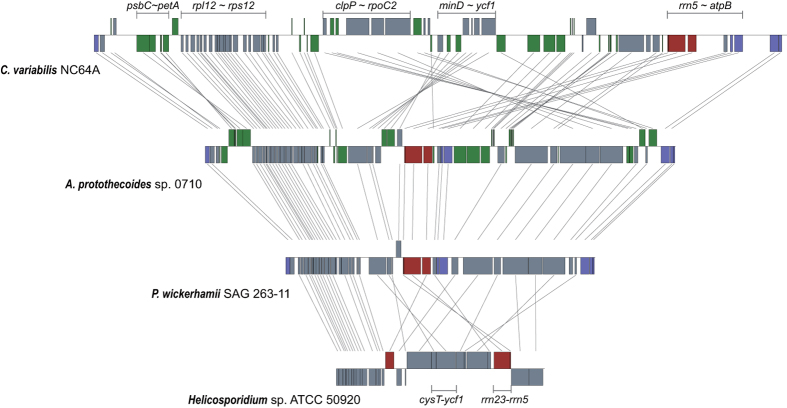
Gene order comparisons between trebouxiophyte plastid genomes. Genomes are drawn to scale. Genes are represented by filled boxes; photosynthetic, ATP synthase and rRNA-encoding genes are indicated by green, blue and red boxes, respectively. Identical genes between the genomes are connected by straight lines.

The *A. protothecoides* ptDNA encodes 76 proteins, 3 rRNAs, and 30 tRNAs, which is among the lowest plastid gene contents currently found in green algae (Table 1; Supplementary Table S3). Not surprisingly, given its non-photosynthetic existence, the *P. wickerhamii* plastid genome encodes even fewer gene products than *A. protothecoides*—40 proteins, 3 rRNAs, and 27 tRNAs. All of the genes in the ptDNA of *P. wickerhamii* are also present in that of *A. protothecoides* (Supplementary Tables S1 and S2). In both *A. protothecoides* and *P. wickerhamii*, most of the ptDNA genes have the same transcriptional polarity, and, more importantly, the gene orders are highly conserved between these two algae (Fig. 1). Such a high degree of similarity in plastid gene arrangement is rarely observed between photosynthetic and non-photosynthetic species. *A. protothecoides* and *P. wickerhamii* show fewer regions of plastid gene collinearity with other trebouxiophytes, such as *C. variablis* and *Helicosporidium* sp., as they do with each other (Fig. 2). Pairwise plastid-gene-order comparisons using a broader sampling of green algae (Supplementary Figure S1 and S2) further supports the hypothesis that the ptDNA synteny between *A. protothecoides* and *P. wickerhamii* is among the highest yet observed within the Chlorophyta, when comparing species from distinct lineages.

### The presence and absence of photosynthesis-related genes in the *A. protothecoides* and *P. wickerhamii* ptDNAs

*A. protothecoides* and *P. wickerhamii* have very different modes of energy production—the former is photosynthetic whereas the latter is an obligate heterotroph. Therefore, it is unexpected that various phylogenetic analyses showed that *P. wickerhamii* is more closely related to *A. protothecoides* than to *Helicosporidium* spp., implying that the loss of photosynthesis has occurred at least twice in the Chlorellales^11^: once in the lineage giving rise to *Helicosporidium* and once within that giving rise to *Prototheca*.

**Figure 3:**
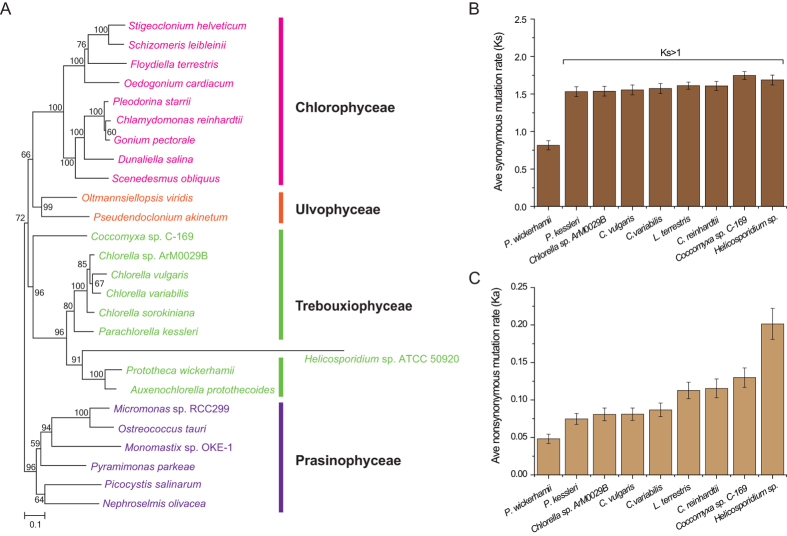
Phylogenetic niche of *A. protothecoides* as inferred from plastid gene sequences and average plastid mutation rates within the Chlorophyta. (**A**) The best Maximum Likelihood phylogenetic tree computed with PHYML under the LG + G8 + I model of amino acid substitution is shown here, with prasinophytes used as outgroups. Bootstrap support for each clade is indicated on the corresponding node. (**B**) Average synonymous mutation rate (Ks) among *A. protothecoides* and nine related species. (**C**) Average non-synonymous mutation rate (Ka) among *A. protothecoides* and nine related species.

To gain more insight into the evolutionary relationships among *A. protothecoides, P. wickerhamii*, and other algae, we performed a Maximum-likelihood phylogenetic analysis, using peptide sequences from 12 single-copy plastid-encoded proteins from 26 species from throughout the Chlorophyta (Fig. 3A). The resulting tree placed *A. protothecoides*, *P. wickerhamii* and *Helicosporidium* sp. together within a clade adjacent to the one containing *Chlorella* sp. ArM0029B, *C. variabilis*, *C. vulgaris*, *P. kessleri*, *Chlorella sorokiniana*, consistent with the fact that all of these algae belong to Chlorellaceae. Moreover, *A. protothecoides* appears to be most closely (bootstrap support 100%) related to *P. wickerhamii*. The two algae are separated by relative short branch lengths, suggesting that they diverged from one another recently in evolutionary history. These results are consistent well with the phylogenies based on 18 s rRNA (Supplementary Figure S3) and previous phylogenetic analyses using rRNA genes^15^,^23^. In addition, a previous mitochondrial phylogenetic analysis placed *P. wickerhamii* and *Helicosporidium* sp. in the same clade^24^. When we included *Helicosporidium* sp. in the plastid phylogeny we found that it is rather basal to the *A. protothecoides* and *P. wickerhamii* clade, suggesting that it is a close relative of *P. wickerhamii* and *A. protothecoides*.

The rate of synonymous substitution (Ks) can be used to estimate the divergent time among species^25^. The average Ks of plastid genes between *A. protothecoides* and *P. wickerhamii* is 0.816 (Fig. 3B). If we assume that the nuclear mutation rate in unicellular green alga^26^ is 3.23 × 10^−10^ substitution per generation, then the number of generation that occurred since their divergence between *A. protothecoides* and *P. wickerhamii* is about 2.52 × 10^9^. When considering that single-celled algae typically have a short generation time and that they typically have similar mutation rates in their plastid and nuclear genomes^27^,^28^,^29^,^30^, then the predicted time of divergent time for *A. protothecoides* and *P. wickerhamii* should be between six to twenty million years. Further supporting the hypothesis that *A. protothecoides* and *P. wickerhamii* are closely related is the fact that the levels of synonymous substitution in the ptDNA are not saturated (<1) and are in fact similar to those observed between other closely related algal strains or species^27^,^28^,^29^. Furthermore, we calculated the similarity of each gene, as well as the 5′UTR (50 bp upstream of ATG) between *P. wickerhamii ptDNA* and its 17 relatives. We found that the overall similarities between the plastid genomes of *P. wickerhamii* and *A. protothecoides* are highest (Supplementary Figure S4) in all the comparisons. Among them, the tRNA genes are more conserved than other coding genes, while the upstream of tRNA genes is more diverged than the coding genes.

We also investigated the various plastid genomic changes that occurred in the *Prototheca* lineage following the loss of photosynthesis. Among 109 genes in the ptDNA of *A. protothecoides* ptDNA, 70 are also present in that of *P. wickerhamii*, meaning 39 genes were lost from the lineage of *P. wickerhamii* following its divergence from that of *A. protothecoides* (Supplementary Table S1 and S2). The majority of the missing genes are related to photosynthesis. For instance, in the *A. protothecoides* plastid genome, 31 genes are involved in photosynthesis, and these genes have been lost from *P. wickerhamii* (and *Helicosporidium* sp.) (Supplementary Table S4 and S5). Three other genes (*ycf3*, *ycf4* and *ycf12*), with ambiguous functions but likely connected to photosynthesis^31^,^32^,^33^, are also absent from the *P. wickerhamii* ptDNA, as are *cemA* and *ccsA*, which encode a plastid envelope membrane protein^34^ and cytochrome c-type biogenesis protein^35^, respectively. Finally, three tRNAs, (trn*L*(GAG)), *trnS*(GGA) and *trnT*(GGU)) have also been eliminated from the *P. wickerhamii* plastid. Significant gene content differences were also observed between *P. wickerhamii* and *Helicosporidium* sp. (Fig. 2, Supplementary Table S3 and S4), indicating a more complex metabolism in *P. wickerhamii’*s plastid compared with that predicted to be located in the plastid of *Helicosporidium* sp.^36^.

To investigate the molecular mechanisms of plastid gene loss from *P. wickerhamii*, we analyzed the junctions flanking deleted genes and gene clusters relative to *A. protothecoides*. In total, 17 regions containing missing genes were identified (labeled breakpoint BP 1 to BP17), ranging from <0.5 kb to >7.5 kb (Supplementary Table S6 and Supplementary Figure S5). Most of the junctions show no sequence similarity, but they do tend to be very AT rich (average >85%), and 13 of the 17 BPs are adjacent to a tRNA gene.

We compared in detail the ptDNAs of *P. wickerhamii* and *Helicosporidium* sp., and found that although both genomes are reduced, the overall architecture are quite different. In the *Helicosporidium* ptDNA, the rRNA operon is split, and the coding regions display a symmetric strand bias^8^. In contrast, the *P. wickerhamii* plastid has a “typical” intact rRNA operon and the coding sequences have an asymmetric strand bias—almost all genes are transcribed in one direction (Fig. 1).

## Discussion

The ptDNAs of non-photosynthetic species are generally <80 kb, making them much smaller than those of most photosynthetic plants and algae, which are about 100–200 kb^37^, with some notable exceptions^38^. In this study, we showed that the *A. protothecoides* ptDNA is among the smallest observed from photosynthetic algae, particularly those from the Trebouxiophyceae and Chlorophyceae. Moreover, the ptDNA architecture and sequence of *A. protothecoides* is similar in many ways to that of its close non-photosynthetic relative *P. wickerhamii* (Figs 1 and 2, Table 1). Indeed, the only significant difference between the ptDNAs of these two algae is the loss of photosynthesis-related genes in the latter. Again, our phylogenetic analyses are consistent with earlier studies showing that *A. protothecoides* is more closely related to *P. wickerhamii* than it is to other species within the Chlorellales^23^, and ultimately support the independent loss of photosynthesis in the *P. wickerhamii* and *Helicosporidium* lineages.

*A. protothecoides* is a free-living mixotrophic alga and, thus, can survive heterotrophically, provided it has organic carbon sources—a feature that has been exploited to produce large amount of biomass in a short period of time^20^. *P. wickerhamii*, on the other hand, is a widely distributed, obligate heterotrophic alga, that can act as an opportunistic pathogens to infect humans^39^ and animals^40^. Although these two algae could not have more drastically different lifestyles^41^,^42^, we found that both share a surprisingly high level of ptDNA sequence identity, which explains that *A. protothecoides* and *P. wickerhamii* are more closely related to one another than they are to other members of the *Chlorella* and *Prototheca* genera, and validates previous results using phylogeny^15^.

The major difference between the *A. protothecoides* and *P. wickerhamii* ptDNA is the presence of photosynthesis-related genes. Given the high similarity in gene content and genomic structure between *A. protothecoides* and *P. wickerhamii*, it is almost certain that they share a recent common photosynthetic ancestor, perhaps as recently as ~6 million years ago. At some point after the two lineages diverged, photosynthesis was lost in the *P. wickerhamii* lineage, resulting in the wholesale loss of photosynthetic genes, whereas in the *A. protothecoides* lineage photosynthesis has been maintained. Ultimately, the close relationship between the two algae suggests that they could represent an excellent duo for studying the evolutionary loss of photosynthesis.

## Methods

### Strains and Cultivation Conditions

The *A. protothecoides* strain, the medium and cultivation methods used in this study have been described previously^43^. The *P. wickerhamii* strain (SAG 263-11) was purchased from the Culture Collection of Algae at the University of Göttingen, Germany (SAG) and cultured in malt peptone medium containing 10 g/L malt extract (Sigma) and 2.5 g/L proteose-peptone (Sigma).

### Organelle genome sequencing and annotation

The complete *P. wickerhamii* plastid genome was obtained using Sanger chemistry and assembled with the SeqMan program from the DNASTAR Lasergene package. The sequencing primers were designed based on the partially sequenced genome (GenBank: AJ245645.1 and AJ236874.1^13^). To close the gaps, a set of primers was designed based on the conserved genes found in both *A. protothecoide*s and *Helicosporidiium* sp. The *A. protothecoides* plastid genome was obtained as part of the whole genome-sequencing project^18^ (GenBank: APJO01001039.1 and APJO01001000.1). All primer sequences are available upon request.

Both the *A. protothecoides* and *P. wickerhamii* plastid genomes were annotated using the same methods. The gene sets were originally annotated by Dogma (Dual Organellar GenoMe Annotator)^44^ and then curated manually. Protein-coding genes and non-coding RNAs with percent identity lower than 25 and 80 respectively were cut off (E-value 1e-5). Protein-coding genes were examined by Blastx searches against the NCBI non-redundant protein (nr) database and their boundaries adjusted manually whereas tRNA-coding genes were identified by tRNAscan-SE 1.23^45^ using organelle parameters. The complete sequences of the *A. protothecoides* and *P. wickerhamii* plastid genomes have been deposited into GenBank under the access numbers KC843975 (*A. protothecoides*) and KJ001761 (*P. wickerhamii*).

### Pairwise alignment and comparison

The pairwise alignments were performed by Blastn (E-value <= 1e-05) in bl2seq 2.2.23^46^ with ‘−1’ as a penalty for a nucleotide mismatch. Each hit was used to estimate the average identity for all alignments or in 100bp-long windows. In gene order comparisons, protein-coding gene and rRNA gene were considered and identified by gene name. *C. variabilis* NC64A and *Helicosporidium* plastid genomes were adjusted for comparison correspondingly. *C. variabilis* NC64A was reversed and started with *atpI*, while *Helicosporidium* sp. started with *rpl12*.

For mutation rate analyses, we did pairwise alignments using GeneWise for each orthologous gene^47^. The software YN00 in the package PAML 4.8a was used to estimate the synonymous and non-synonymous mutation rate (KS&KA)^48^. Li’s model^49^ was used for estimating DNA divergence time (the generation times using for calculation were 24 h = 0.00274y for heterotroph and 72 h = 0.008219y for autotroph).

### Phylogenetic tree construction

We used the TreeFam methodology^50^ to define orthologous genes among taxa as follows: the all-versus-all peptide sequence alignments of protein-coding genes were performed using blastp 2.2.23 (no SEG query sequence filtering, *E*-value threshold of 1e-7), the blastp alignments were combined and filtered using SOLAR 0.9.6 (pairwise gene alignment rate of 0.24), and the clustering was performed with Hcluster_sg 0.5.0 (minimum edge weight 10, minimum edge density 0.34).

A total of 12 single-copy TreeFam protein-encoding gene clusters (*tufA*, *rpoC1*, *rps7*, *rps8*, *rps11*, *rps12*, *rps19*, *rpl2*, *rpl5*, *rpl14*, *rpl16*, *rpl20*) from 26 Chlorophyta taxa were defined. The amino acid sequences were aligned with MUSCLE 3.7^51^ and the ambiguously aligned regions were filtered out with BMGE 1.1^52^ using the default parameters. Maximum-Likelihood phylogenetic inferences were run with PhyML 3.0^53^ under the LG + G + I model of amino acid substitution (8 gamma categories), selected with the Model Selection (ML) module from MEGA 6.05^54^. A total of 100 non-parametric bootstrap replicates were performed, as implemented in PHYML.

## Additional Information

**How to cite this article**: Yan, D. *et al. Auxenochlorella protothecoides* and *Prototheca wickerhamii* plastid genome sequences give insight into the origins of non-photosynthetic algae. *Sci. Rep.* **5**, 14465; doi: 10.1038/srep14465 (2015).

## Supplementary Material

Supplementary Information

## Figures and Tables

**Table 1:** General features of known ptDNAs in Trebouxiophytes.

Species	Size (bp)	%ncDNA (including introns)a,^b^	Mean intergenic distance (bp)	Protein^c^	rRNA^d^	tRNA	G + C (%)	Inverted repeats	Accession
*Helicosporidium* sp.	37,454	22.2	319	26	3	25	26.9		NC_008100
*Prototheca wickerhamii*	55,636	28.8	400	40	3	27	31.2		KJ001761
*Auxenochlorella protothecoides*	84,580	24.6	273	76	3	30	30.8		KC843975
*Chlorella sorokiniana*	109,811	43.0	629	75	3	31	34.0		NC_023835
*Chlorella* sp. ArM0029B	119,989	46.2	692	80	3	32	33.9		KF554427
*Parachlorella kessleri*	123,994	45.3	668	84	6	36	30.0	10,913	NC_012978
*Chlorella variabilis*	124,579	49.5	770	80	3	32	33.9		NC_015359
*Trebouxiophyceae* sp. MX-AZ01	149,707	52.2	977	80	3	32	57.7		NC_018569
*Chlorella vulgaris*	150,613	43.3	375	174	3	33	31.6		NC_001865
*Coccomyxa* sp. C-169	175,731	60.0	1318	80	3	32	50.7		NC_015084
*Leptosira terrestris*	195,081	56.8	1259	88	3	28	27.3		NC_009681

^a^Conserved genes, unique ORFs and intronic ORFs were counted as coding sequences.

^b^ncDNA; non-coding DNA.

^c,d^Genes within inverted repeats were counted twice.

## References

[b1] Gould S. B., Waller R. F. & McFadden G. I. Plastid evolution. Annu Rev Plant Biol 59, 491–517 (2008). 18315522 10.1146/annurev.arplant.59.032607.092915

[b2] Gross J. & Bhattacharya D. Mitochondrial and plastid evolution in eukaryotes: an outsiders’ perspective. Nat Rev Genet 10, 495–505 (2009). 19506574 10.1038/nrg2610

[b3] Keeling P. J. The endosymbiotic origin, diversification and fate of plastids. Philos Trans R Soc Lond B Biol Sci 365, 729–748 (2010). 20124341 10.1098/rstb.2009.0103 PMC2817223

[b4] Williams B. A. & Hirt R. P. RACE and RAGE cloning in parasitic microbial eukaryotes. Methods Mol Biol 270, 151–172 (2004). 15153626 10.1385/1-59259-793-9:151

[b5] Krause K. From chloroplasts to “cryptic” plastids: evolution of plastid genomes in parasitic plants. Curr Genet 54, 111–121 (2008). 18696071 10.1007/s00294-008-0208-8

[b6] Smith D. R. & Lee R. W. A plastid without a genome: evidence from the nonphotosynthetic green algal genus Polytomella. Plant Physiol 164, 1812–1819 (2014). 24563281 10.1104/pp.113.233718 PMC3982744

[b7] Turmel M., Pombert J. F., Charlebois P., Otis C. & Lemieux C. The Green Algal Ancestry of Land Plants as Revealed by the Chloroplast Genome. International Journal of Plant Sciences 168, 679–689 (2007).

[b8] de Koning A. P. & Keeling P. J. The complete plastid genome sequence of the parasitic green alga *Helicosporidium* sp. is highly reduced and structured. BMC Biol 4, 12 (2006). 16630350 10.1186/1741-7007-4-12 PMC1463013

[b9] Gockel G. & Hachtel W. Complete gene map of the plastid genome of the nonphotosynthetic euglenoid flagellate *Astasia longa*. Protist 151, 347–351 (2000). 11212895 10.1078/S1434-4610(04)70033-4

[b10] Molina J. *et al.* Possible loss of the chloroplast genome in the parasitic flowering plant Rafflesia lagascae (Rafflesiaceae). Mol Biol Evol 31, 793–803 (2014). 24458431 10.1093/molbev/msu051 PMC3969568

[b11] Figueroa-Martinez F., Nedelcu A. M., Smith D. R. & Reyes-Prieto A. When the lights go out: the evolutionary fate of free-living colorless green algae. New Phytologist n/a-n/a (2015). 10.1111/nph.13279 PMC5024002 26042246

[b12] Tartar A. The Non-Photosynthetic Algae Helicosporidium spp.: Emergence of a Novel Group of Insect Pathogens. Insects 4, 375–391 (2013). 10.3390/insects4030375 PMC4553470 26462425

[b13] Tartar A., Boucias D. G., Becnel J. J. & Adams B. J. Comparison of plastid 16S rRNA (*rrn16*) genes from *Helicosporidium* spp.: evidence supporting the reclassification of Helicosporidia as green algae (Chlorophyta). Int J Syst Evol Microbiol 53, 1719–1723 (2003). 14657099 10.1099/ijs.0.02559-0

[b14] Knauf U. & Hachtel W. The genes encoding subunits of ATP synthase are conserved in the reduced plastid genome of the heterotrophic alga *Prototheca wickerhamii*. Mol Genet Genomics 267, 492–497 (2002). 12111556 10.1007/s00438-002-0681-6

[b15] Ueno R., Urano N. & Suzuki M. Phylogeny of the non-photosynthetic green micro-algal genus *Prototheca* (Trebouxiophyceae, Chlorophyta) and related taxa inferred from SSU and LSU ribosomal DNA partial sequence data. FEMS Microbiol Lett 223, 275–280 (2003). 12829298 10.1016/S0378-1097(03)00394-X

[b16] Ewing A. *et al.* 16S and 23S plastid rDNA phylogenies of species and their auxanographic phenotypes. J Phycol 50, 765–769 (2014). 25937672 10.1111/jpy.12209 PMC4373152

[b17] Darienko T. & Proschold T. Genetic Variability And Taxonomic Revision Of the Genus Auxenochlorella (Shihira Et Krauss) Kalina Et Puncocharova (Trebouxiophyceae, Chlorophyta). Journal Of Phycology 51, 394–400 (2015). 10.1111/jpy.12279 26986533

[b18] Champenois J., Marfaing H. & Pierre R. Review of the taxonomic revision of Chlorella and consequences for its food uses in Europe. Journal of Applied Phycology (2014).

[b19] Shi X. M. & Chen F. Production and rapid extraction of lutein and the other lipid-soluble pigments from *Chlorella protothecoides* grown under heterotrophic and mixotrophic conditions. Food/Nahrung 43, 109–113 (1999).

[b20] Miao X. & Wu Q. Biodiesel production from heterotrophic microalgal oil. Bioresour Technol 97, 841–846 (2006). 15936938 10.1016/j.biortech.2005.04.008

[b21] Xiong W., Gao C., Yan D., Wu C. & Wu Q. Double CO_2_ fixation in photosynthesis-fermentation model enhances algal lipid synthesis for biodiesel production. Bioresour Technol 101, 2287–2293 (2010). 19963369 10.1016/j.biortech.2009.11.041

[b22] Gao C. *et al.* Oil accumulation mechanisms of the oleaginous microalga *Chlorella protothecoides* revealed through its genome, transcriptomes, and proteomes. BMC Genomics 15, 582 (2014). 25012212 10.1186/1471-2164-15-582 PMC4111847

[b23] Blanc G. *et al.* The *Chlorella variabilis* NC64A genome reveals adaptation to photosymbiosis, coevolution with viruses, and cryptic sex. Plant Cell 22, 2943–2955 (2010). 20852019 10.1105/tpc.110.076406 PMC2965543

[b24] Smith D. R. *et al.* The GC-rich mitochondrial and plastid genomes of the green alga *Coccomyxa* give insight into the evolution of organelle DNA nucleotide landscape. PLoS One 6, e23624 (2011). 21887287 10.1371/journal.pone.0023624 PMC3162594

[b25] Nei M. & Kumar S. Molecular evolution and phylogenetics. 52–256 (Oxford University Press, 2000).

[b26] Ness R. W., M. A., Colegrave N. & Keightley P. D. Estimate of the spontaneous mutation rate in *Chlamydomonas reinhardtii*. Genetics 192, 1447–1454 (2012). 23051642 10.1534/genetics.112.145078 PMC3512149

[b27] Hua J., Smith D. R., Borza T. & Lee R. W. Similar relative mutation rates in the three genetic compartments of Mesostigma and Chlamydomonas. Protist 163, 105–115 (2012). 21621456 10.1016/j.protis.2011.04.003

[b28] Smith D. R., Arrigo K. R., Alderkamp A. C. & Allen A. E. Massive difference in synonymous substitution rates among mitochondrial, plastid, and nuclear genes of Phaeocystis algae. Molecular phylogenetics and evolution 71, 36–40 (2014). 24216019 10.1016/j.ympev.2013.10.018

[b29] Smith D. R., Jackson C. J. & Reyes-Prieto A. Nucleotide substitution analyses of the glaucophyte Cyanophora suggest an ancestrally lower mutation rate in plastid vs mitochondrial DNA for the Archaeplastida. Mol Phylogenet Evol 79, 380–384 (2014). 25017510 10.1016/j.ympev.2014.07.001

[b30] Santos C. *et al.* Mutation patterns of mtDNA: empirical inferences for the coding region. BMC Evol Biol 8, 167 (2008). 18518963 10.1186/1471-2148-8-167 PMC2438339

[b31] Boudreau E., Takahashi Y., Lemieux C., Turmel M. & Rochaix J. D. The chloroplast ycf3 and ycf4 open reading frames of *Chlamydomonas reinhardtii* are required for the accumulation of the photosystem I complex. EMBO J 16, 6095–6104 (1997). 9321389 10.1093/emboj/16.20.6095 PMC1326293

[b32] Naver H., Boudreau E. & Rochaix J. D. Functional studies of Ycf3: its role in assembly of photosystem I and interactions with some of its subunits. Plant Cell 13, 2731–2745 (2001). 11752384 10.1105/tpc.010253 PMC139485

[b33] Kashino Y. *et al.* Ycf12 is a core subunit in the photosystem II complex. Biochim Biophys Acta 1767, 1269–1275 (2007). 17935689 10.1016/j.bbabio.2007.08.008

[b34] Katoh A., Lee K. S., Fukuzawa H., Ohyama K. & Ogawa T. *cemA* homologue essential to CO_2_ transport in the cyanobacterium *Synechocystis* PCC6803. Proc Natl Acad Sci USA 93, 4006–4010 (1996). 8633006 10.1073/pnas.93.9.4006 PMC39476

[b35] Feissner R. E., Beckett C. S., Loughman J. A. & Kranz R. G. Mutations in cytochrome assembly and periplasmic redox pathways in *Bordetella pertussis*. J Bacteriol 187, 3941–3949 (2005). 15937156 10.1128/JB.187.12.3941-3949.2005 PMC1151747

[b36] Borza T., Popescu C. E. & Lee R. W. Multiple metabolic roles for the nonphotosynthetic plastid of the green alga *Prototheca wickerhamii*. Eukaryot Cell 4, 253–261 (2005). 15701787 10.1128/EC.4.2.253-261.2005 PMC549340

[b37] Barbrook A. C., Howe C. J. & Purton S. Why are plastid genomes retained in non- photosynthetic organisms? Trends Plant Sci 11, 101–108 (2006). 16406301 10.1016/j.tplants.2005.12.004

[b38] Del Vasto M. *et al.* Massive and widespread organelle genomic expansion in the green algal genus dunaliella. Genome biology and evolution 7, 656–663 (2015). 25663488 10.1093/gbe/evv027 PMC5322560

[b39] Kantrow S. M. & Boyd A. S. Protothecosis. Dermatol Clin 21, 249–255 (2003). 12757247 10.1016/s0733-8635(02)00089-x

[b40] Hollingsworth S. R. Canine protothecosis. Vet Clin North Am Small Anim Pract 30, 1091–1101 (2000). 11033876 10.1016/s0195-5616(00)05008-7

[b41] Huss V. A. R., Frank C., Hartmann E., Hirmer M., Kloboucek A., Seidel B. M., Wenzeler P. & Kessler E. Biochemical taxonomy and molecular phylogeny of the genus *Chlorella* sensu lato (Chlorophyta). J Phycol 35, 587–598 (1999).

[b42] Ueno R., Hanagata N., Urano N. & Suzuki M. Molecular phylogeny and phenotypic variation in the heterotrophic green algal genus *Prototheca* (Trebouxiophyceae, Chlorophyta). J Phycol 41, 1268–1280 (2005).

[b43] Yan D., Lu Y., Chen Y. F. & Wu Q. Waste molasses alone displaces glucose-based medium for microalgal fermentation towards cost-saving biodiesel production. Bioresour Technol 102, 6487–6493 (2011). 21474303 10.1016/j.biortech.2011.03.036

[b44] Wyman S. K., Jansen R. K. & Boore J. L. Automatic annotation of organellar genomes with DOGMA. Bioinformatics 20, 3252–3255 (2004). 15180927 10.1093/bioinformatics/bth352

[b45] Lowe T. M. & Eddy S. R. tRNAscan-SE: a program for improved detection of transfer RNA genes in genomic sequence. Nucleic Acids Res 25, 955–964 (1997). 9023104 10.1093/nar/25.5.955 PMC146525

[b46] Tatusova T. A. & Madden T. L. BLAST 2 Sequences, a new tool for comparing protein and nucleotide sequences. FEMS Microbiol Lett 174, 247–250 (1999). 10339815 10.1111/j.1574-6968.1999.tb13575.x

[b47] Birney E., Clamp M. & Durbin R. GeneWise and Genomewise. Genome Res 14, 988–995 (2004). 15123596 10.1101/gr.1865504 PMC479130

[b48] Yang Z. & Nielsen R. Estimating synonymous and nonsynonymous substitution rates under realistic evolutionary models. Mol Biol Evol 17, 32–43 (2000). 10666704 10.1093/oxfordjournals.molbev.a026236

[b49] Li W. Molecular Evolution (MA: Sinauer Associates, 1997).

[b50] Li H. *et al.* TreeFam: a curated database of phylogenetic trees of animal gene families. Nucleic Acids Res 34, D572–580 (2006). 16381935 10.1093/nar/gkj118 PMC1347480

[b51] Edgar R. C. MUSCLE: multiple sequence alignment with high accuracy and high throughput. Nucleic Acids Res 32, 1792–1797 (2004). 15034147 10.1093/nar/gkh340 PMC390337

[b52] Criscuolo A. & Gribaldo S. BMGE (Block Mapping and Gathering with Entropy): a new software for selection of phylogenetic informative regions from multiple sequence alignments. BMC Evol Biol 10, 210 (2010). 20626897 10.1186/1471-2148-10-210 PMC3017758

[b53] Guindon S. *et al.* New algorithms and methods to estimate maximum- likelihood phylogenies: assessing the performance of PhyML 3.0. Syst Biol 59, 307–321 (2010). 20525638 10.1093/sysbio/syq010

[b54] Tamura K., Stecher G., Peterson D., Filipski A. & Kumar S. MEGA6: Molecular Evolutionary Genetics Analysis version 6.0. Mol Biol Evol 30, 2725–2729 (2013). 24132122 10.1093/molbev/mst197 PMC3840312

