# Erratum: *Auxenochlorella protothecoides* and *Prototheca wickerhamii* plastid genome sequences give insight into the origins of non-photosynthetic algae

**DOI:** 10.1038/srep17211

**Published:** 2015-12-14

**Authors:** Dong Yan, Yun Wang, Tatsuya Murakami, Yue Shen, Jianhui Gong, Huifeng Jiang, David R. Smith, Jean-Francois Pombert, Junbiao Dai, Qingyu Wu

Scientific Reports 5: Article number: 1446510.1038/srep14465; published online: 09252015; updated: 12142015

The Acknowledgements section in this Article is incomplete.

“This work was supported by MOST project 2011CB808804 and 2014AA02200, NSFC project 31370282 and 41030210 to Q.W. and by Tsinghua University Initiative Scientific Research Program 2011Z02296 and 2012Z08128 to J.D.”

should read:

“This work was supported by MOST project 2011CB808804 and 2014AA02200, NSFC project 31370282 and 41030210 to Q.W. and by Tsinghua University Initiative Scientific Research Program 2011Z02296 and 2012Z08128 to J.D., and partially by MOST project 2012AA02A701.

