# Supplementary material for: Auxenochlorella protothecoides and Prototheca wickerhamii plastid genome sequences give insight into the origins of non-photosynthetic algae
Source: Sci Rep. 2015 Sep 25;5:14465. doi: 10.1038/srep14465 (PMC4585924; doi:10.1038/srep14465)
Supplement: Supplementary Information [file srep14465-s1.doc]

*Supplementary Figures and Tables*

***Auxenchlorella protothecoides* and *Prototheca wickerhamii* plastid genome sequences give insight into the origins of non-photosynthetic algae**

Dong Yan^1,#,$^, Yun Wang^2,#^, Tatsuya Murakami^1^, Yue Shen^2^, Jianhui Gong^2^, Huifeng Jiang^3^, David R. Smith^4^, Jean-Francois Pombert^5^, Junbiao Dai^1,*^ , Qingyu Wu^1,*^

^1^MOE Key Laboratory of Bioinformatics and Center for Synthetic and System Biology, Tsinghua University, Beijing 100084, China

^2^BGI-Shenzhen, Shenzhen 518083, China

^3^Key Laboratory of Systems Microbial Biotechnology, Tianjin Institute of Industrial Biotechnology, Chinese Academy of Sciences, Tianjing 300308, China

^4^Department of Biology, University of Western Ontario, London, Ontario, N6A 5B7, Canada

^5^College of Science, Illinois Institute of Technology, Chicago, IL 60616, USA

^#^These authors contributed equally to this work.

$Current address: Department of Mechanical Engineering, Tsinghua University, Beijing 100084, China

***Corresponding authors:**

Dr. Junbiao Dai, Center for Epigenetics and Chromatin, School of Life Sciences, Tsinghua University, Beijing 100084, China; Phone: +86-10-62796190; Fax: +86-10-62796190; E-mail: [jbdai@tsinghua.edu.cn](mailto:jbdai@tsinghua.edu.cn).

Dr. Qingyu Wu, School of Life Sciences, Tsinghua University, Beijing 100084, China; Phone: +86-10-62781825; Fax: +86-10-62781825; E-mail: [qingyu@mail.tsinghua.edu.cn](mailto:qingyu@mail.tsinghua.edu.cn)


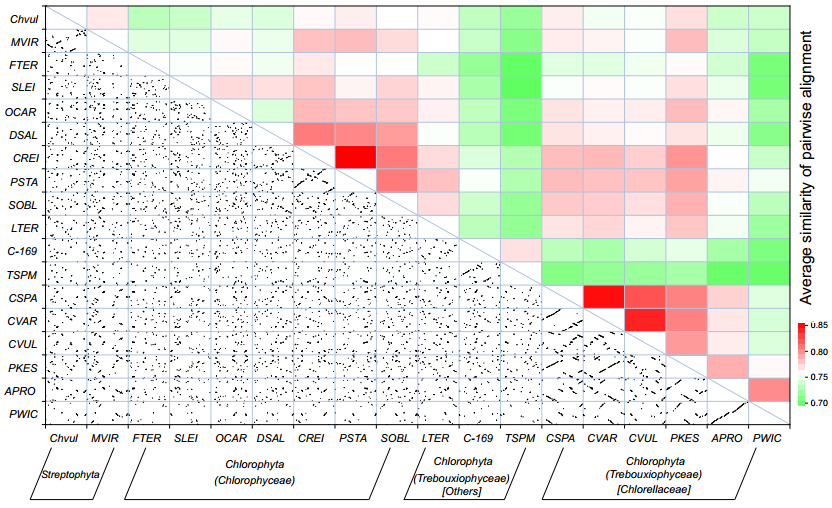


**Figure S1 | Pairwise alignments of chloroplast/plastid genomes** **among *C. protothecoides sp. 0710* and its relatives.** The pairwise alignments were performed by Blastn (E-value <= 1e-05) in bl2seq 2.2.23 with '-1' as a penalty for a nucleotide mismatch. The upper half (heat map) shows the average identity for all the pairwise alignments, and the median (Me=0.758) indicated in white. The lower half (dot-plots) shows each pairwise alignment. Only the hits longer than 400 bp are shown here (each dot spans 200 bp). *Chvul*: *Chara vulgaris*; *MVIR*: *Mesostigma viride*; *FTER*: *Floydiella terrestris*; *SLEI*: *Schizomeris leibleinii*; *OCAR*: *Oedogonium cardiacum*; *DSAL*: *Dunaliella salina*; *CREI*: *Chlamydomonas reinhardtii*; *PSTA*: *Pleodorina starrii*; *SOBL*: *Scenedesmus obliquus*; *LTER*: *Leptosira terrestris*; *C-169*: *Coccomyxa* sp. C-169; *TSPM*: *Trebouxiophyceae* sp. MX-AZ01; *CSPA*: *Chlorella* sp. ArM0029B; *CVAR*: *Chlorella variabilis*; *CVUL*: *Chlorella vulgaris*; *PKES*: *Parachlorella kessleri*; APRO: *A. protothecoides* sp. 0710; *PWIC*: *Prototheca wickerhamii*.


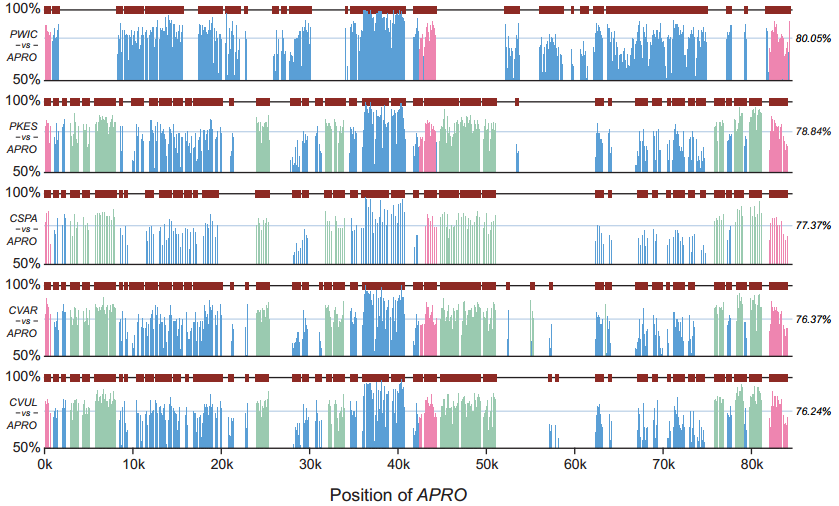


**Figure S2 | Segmental collinearity and completely alignment of the plastid genomes of *A. protothecoides* and five algae in Chlorellaceae.** Here, the complete ptDNA of *A. protothecoides* is compared with every other ptDNA respectively. The pairwise alignments were performed by Blastn (E-value <= 1e-05) in bl2seq 2.2.23 with '-1' as a penalty for a nucleotide mismatch. Each hit was used to estimate the average identity in 100bp-long windows. The grey line and the number on the right represent the average identity for the alignments. Genes related to photosynthesis are indicated in green. Genes related to ATP synthases are indicated in carmine and the remaining ones are indicated in blue. The brown boxes represent each alignment. APRO: *A. protothecoides* sp. 0710, PWIC: *P. wickerhamii*, PKES*: Parachlorella kessleri,* CSPA: *Chlorella* sp. ArM0029B, CVAR: *C. variabilis* NC64A; CVUL: *C. vulgaris.*





**Figure S3 | Phylogenetic tree of *A. protothecoides* and *P. wickerhamii* within 58**
**taxas as inferred from 18s rRNA sequences.**

The phylogenetic tree was inferred by the maximum likelihood method based on a data set of 1,376 aligned positions of 58 taxa using phyml v3.0. GTR model(G= 0.466; I= 0.326; nucleotides frequencies: A 0.25728, C 0.21177, G 0.28441, T 0.24654; relative rate parameters: A-C 1.10079, A-G 2.29371, A-T 0.90082, C-G 1.02208, C-T 5.48575, G-T 1.00000) was applied, which is selected by jmodeltest v2.16. The branches in bold were highly supported (bootstrap values >70%). The *A. protothecoides* and *P. wickerhamii* strains used were marked with asterisk.

**Figure S4 | Sequence similarity between *A. protothecoides* and *P. wickerhamii* plastid genome.**

Four panels of boxplots displayed the similarity distributions of mRNA, 5’UTR of mRNA, tRNA and 5’UTR of tRNA between *P. wickerhamii*  and its 17 relatives by Clustal-omega, respectively. The order of boxplots in a panel is according to the increasing of average similarity over all genes. The distinct color is roughly set to represent different groups.


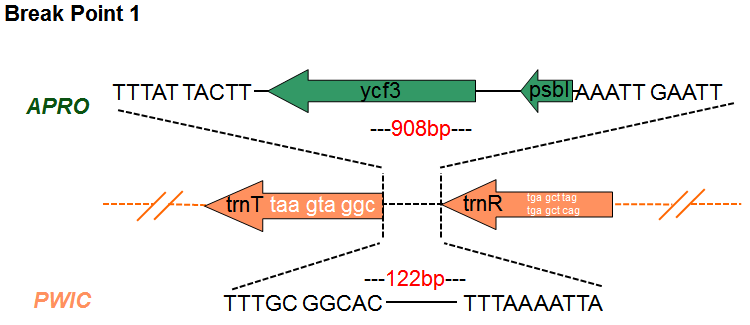

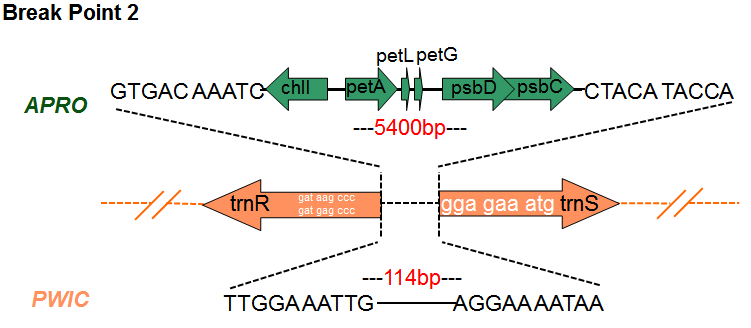

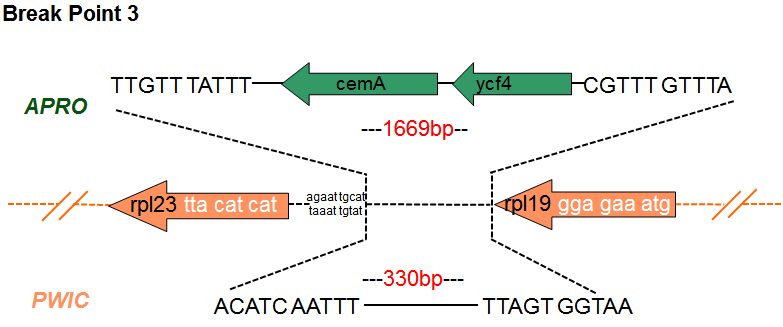

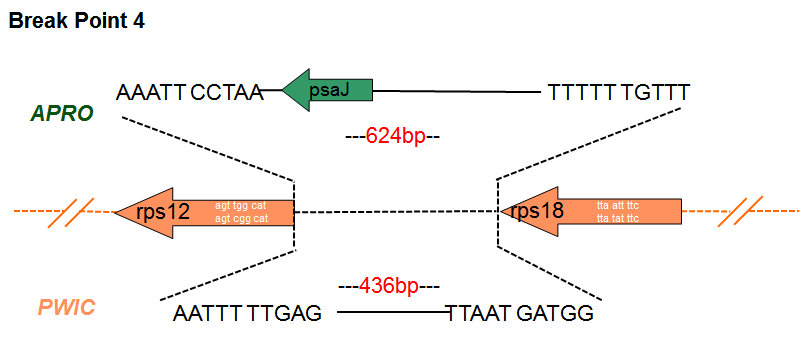


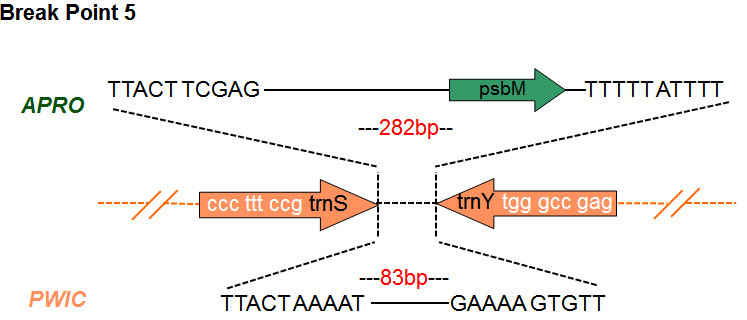

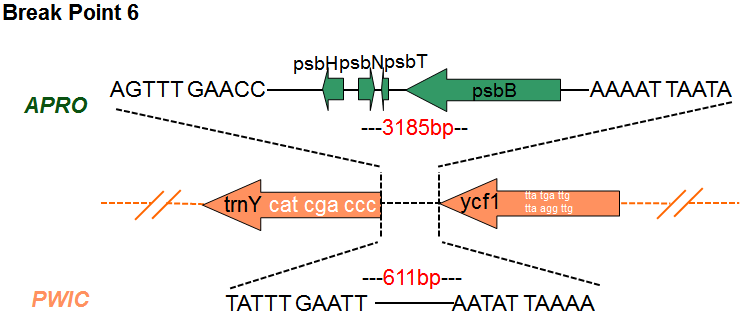

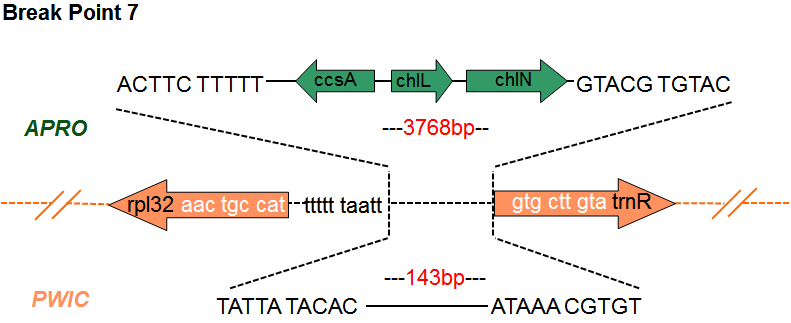

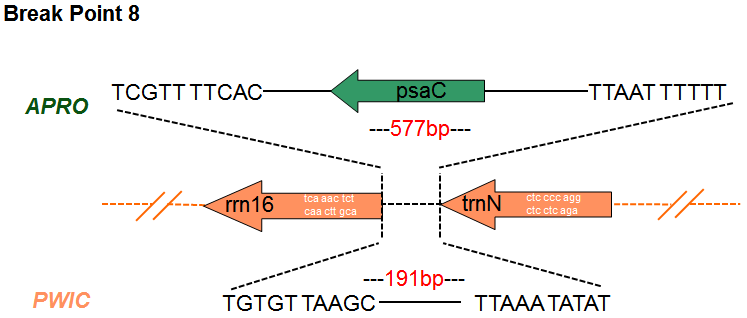


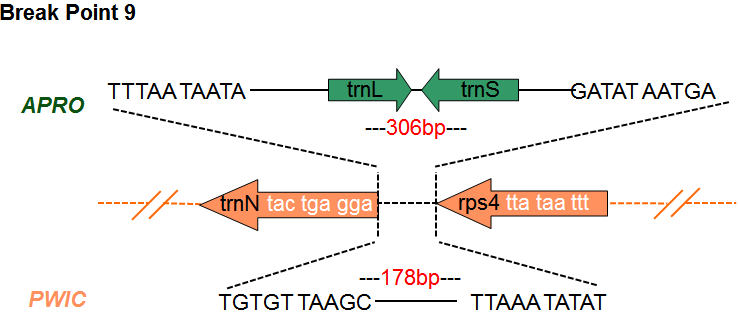

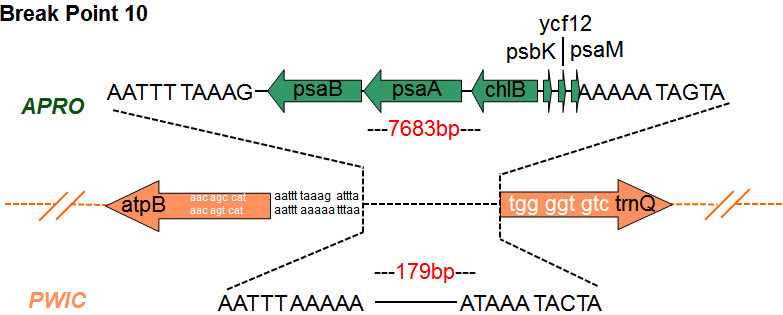

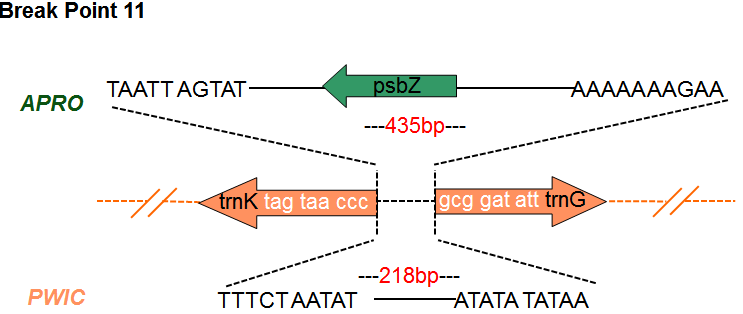

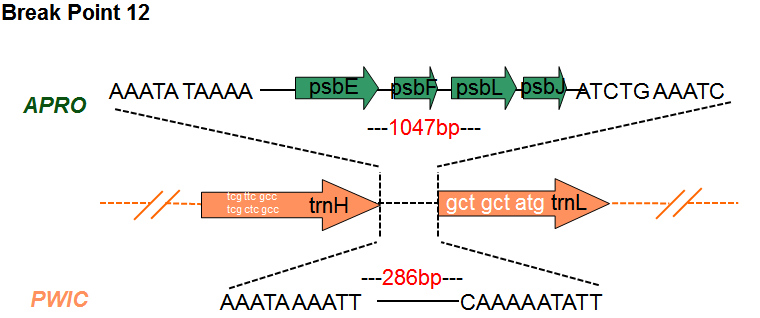


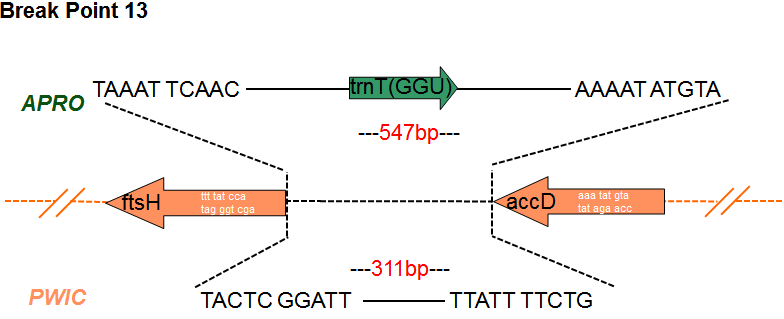

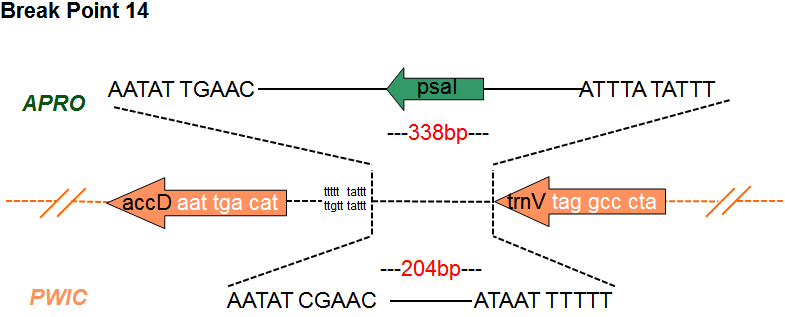

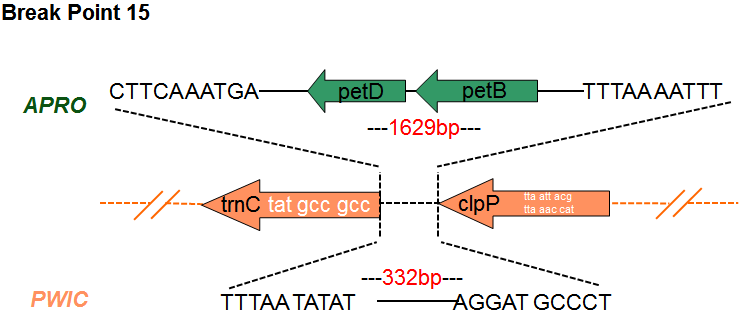

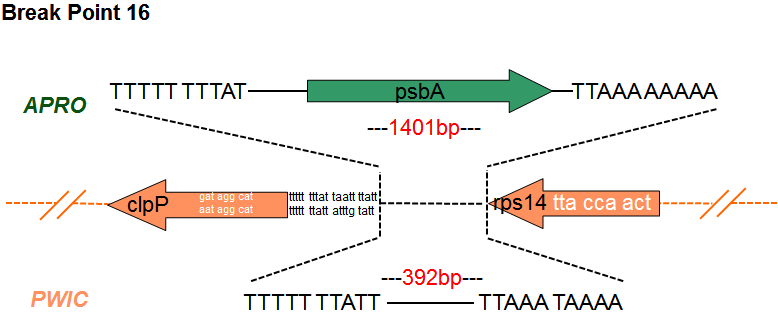


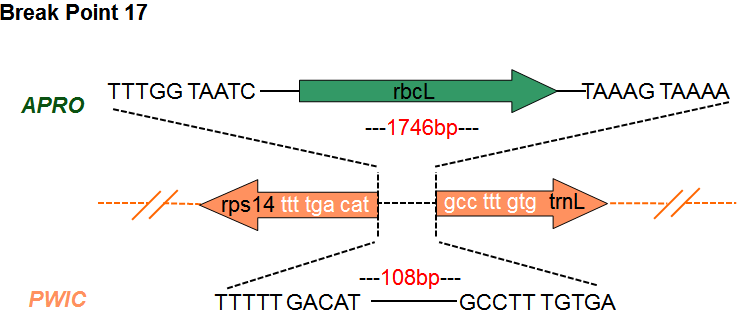


**Figure S5| 17 break point regions containing the missing genes.** Homologous regions (HR) are represented in orange. Only the ten nucleotides adjacent HRs are shown. The size of the variable region is indicated in red**.**

**Supplementary Table S1 | Gene list of *Auxenchlorella protothecoides* plastid DNA**

|  | RNA Genes | | | | | | | | | | | | | | | |  |
| --- | --- | --- | --- | --- | --- | --- | --- | --- | --- | --- | --- | --- | --- | --- | --- | --- | --- |
| Ribosomal RNAs (3) | *23s rRNA* | | *16s rRNA* | | *5s rRNA* | |  | |  | |  | |  | |  | |  |
| Transfer RNAs (30) | *trnA*(UGC) | | *trnC*(GCA) | | *trnD*(GUC) | | *trnE*(UUC) | | *trnF*(GAA) | | *trnG*(GCC) | | *trnG*(UCC) | | *trnH*(GUG) | |  |
|  | *trnI*(GAU) | | *trnK*(UUU) | | *trnL*(CAA) | | *trnL*(GAG) | | *trnL*(UAG) | | *trnM*(CAU)×2 | | *trnfM*(CAU) | | *trnN*(GUU) | |  |
|  | *trnP*(UGG) | | *trnR*(ACG) | | *trnR*(CCG) | | *trnR*(UCU) | | *trnS*(GCU) | | *trnS*(UGA) | | *trnS*(GGA) | | *trnT*(GGU) | |  |
|  | *trnT*(UGU) | | *trnV*(UAC) | | *trnW*(CCA) | | *trnY*(GUA) | | *trnQ*(UUG) | |  | |  | |  | |  |
|  | Protein-Coding Genes | | | | | | | | | | | | | | | |  |
| Photosynthesis (31) |  | |  | |  | |  | |  | |  | |  | |  | |  |
| Photosystem I (6) | *psaA* | | *psaB* | | *psaC* | | *psaI* | | *psaM* | | *psaJ* | |  | |  | |  |
| Photosystem II (15) | *psbA* | | *psbB* | | *psbC* | | *psbD* | | *psbE* | | *psbF* | | *psbH* | | *psbI* | |  |
|  | *psbJ* | | *psbK* | | *psbL* | | *psbM* | | *psbN* | | *psbT* | | *psbZ* | |  | |  |
| Cytochrome b6/f (5) | *petA* | | *petB* | | *petD* | | *petG* | | *petL* | |  | |  | |  | |  |
| Chlorophyll biosynthesis (4) | *chlB* | | *chlI* | | *chlL* | | *chlN* | |  | |  | |  | |  | |  |
| Rubisco | *rbcL* | |  | |  | |  | |  | |  | |  | |  | |  |
| ATP synthesis (6) |  | |  | |  | |  | |  | |  | |  | |  | |  |
| ATP synthase (6) | *atpA* | | *atpB* | | *atpE* | | *atpF* | | *atpH* | | *atpI* | |  | |  | |  |
| Ribosomal proteins (21) |  | |  | |  | |  | |  | |  | |  | |  | |  |
| Large subunit (10) | *rpl2* | | *rpl5* | | *rpl12* | | *rpl14* | | *rpl16* | | *rpl19* | | *rpl20* | | *rpl23* | |  |
|  | *rpl32* | | *rpl36* | |  | |  | |  | |  | |  | |  | |  |
| Small subuni (11) | *rps2* | | *rps3* | | *rps4* | | *rps7* | | *rps8* | | *rps9* | | *rps11* | | *rps12* | |  |
|  | *rps14* | | *rps18* | | *rps19* | |  | |  | |  | |  | |  | |  |
| Transcription/translation (6) |  | |  | |  | |  | |  | |  | |  | |  | |  |
| RNA polymerase (4) | *rpoA* | | *rpoB* | | *rpoC1* | | *rpoC2* | |  | |  | |  | |  | |  |
| Translation (2) | *tufA* | | *infA* | |  | |  | |  | |  | |  | |  | |  |
| Division (2) | *minD* | | *ftsH* | |  | |  | |  | |  | |  | |  | |  |
| Other proteins (6) | *accD* | | *cemA* | | *clpP* | | *ccsA* | | *cysT* | | *tilS* | |  | |  | |  |
| Conserved proteins (4) | *ycf1* | | *ycf3* | | *ycf4* | | *ycf12* | |  | |  | |  | |  | |  |
| ×2 two copies |  |  | |  | |  | |  | |  | |  | |  | |  | |

**Supplementary Table S2 | Gene list of *Prototheca wickerhamii* plastid DNA**

|  | | RNA Genes | | | | | | | | | | | | | | | |  |
| --- | --- | --- | --- | --- | --- | --- | --- | --- | --- | --- | --- | --- | --- | --- | --- | --- | --- | --- |
| Ribosomal RNAs (3) | | *23s rRNA* | | *16s rRNA* | | *5s rRNA* | |  | |  | |  | |  | |  | |  |
| Transfer RNAs (27) | | *trnA*(UGC) | | *trnC*(GCA) | | *trnD*(GUC) | | *trnE*(UUC) | | *trnF*(GAA) | | *trnG*(GCC) | | *trnG*(UCC) | | *trnH*(GUG) | |  |
|  | | *trnI*(GAU) | | *trnK*(UUU) | | *trnL*(CAA) | | *trnL*(UAG) | | *trnM*(CAU)×2 | | *trnfM*(CAU) | | *trnN*(GUU) | | *trnP*(UGG) | |  |
|  | | *trnR*(ACG) | | *trnR*(CCG) | | *trnR*(UCU) | | *trnS*(GCU) | | *trnS*(UGA) | | *trnT*(UGU) | | *trnV*(UAC) | | *trnW*(CCA) | |  |
|  | | *trnY*(GUA) | | *trnQ*(UUG) | |  | |  | |  | |  | |  | |  | |  |
|  | | Protein-Coding Genes | | | | | | | | | | | | | | | |  |
| ATP synthesis (6) | |  | |  | |  | |  | |  | |  | |  | |  | |  |
| ATP synthase (6) | | *atpA* | | *atpB* | | *atpE* | | *atpF* | | *atpH* | | *atpI* | |  | |  | |  |
| Ribosomal proteins (21) | |  | |  | |  | |  | |  | |  | |  | |  | |  |
| Large subunit (10) | | *rpl2* | | *rpl5* | | *rpl12* | | *rpl14* | | *rpl16* | | *rpl19* | | *rpl20* | | *rpl23* | |  |
|  | | *rpl32* | | *rpl36* | |  | |  | |  | |  | |  | |  | |  |
| Small subuni (11) | | *rps2* | | *rps3* | | *rps4* | | *rps7* | | *rps8* | | *rps9* | | *rps11* | | *rps12* | |  |
|  | | *rps14* | | *rps18* | | *rps19* | |  | |  | |  | |  | |  | |  |
| Transcription/translation (6) | |  | |  | |  | |  | |  | |  | |  | |  | |  |
| RNA polymerase (4) | | *rpoA* | | *rpoB* | | *rpoC1* | | *rpoC2* | |  | |  | |  | |  | |  |
| Translation (2) | | *tufA* | | *infA* | |  | |  | |  | |  | |  | |  | |  |
| Division (2) | | *minD* | | *ftsH* | |  | |  | |  | |  | |  | |  | |  |
| Other proteins (4) | | *accD* | | *clpP* | | *cysT* | | *tilS* | |  | |  | |  | |  | |  |
| Conserved proteins (1) | | *ycf1* | |  | |  | |  | |  | |  | |  | |  | |  |
| ×2 two copies |  | |  | |  | |  | |  | |  | |  | |  | |  | |

**Supplementary Table S3 | General features of known ptDNAs in** Chlorophyte other than Trebouxiophytes

|  | Size (bp) | % non-coding DNA (including introns)^a^ | Mean intergenic distance (bp) | Protein^b^ | rRNA^c^ | tRNA | G+C (%) | Inverted repeats | Accession No. |
| --- | --- | --- | --- | --- | --- | --- | --- | --- | --- |
| **Ulvophyceae** |  |  |  |  |  |  |  |  |  |
| *Oltmannsiellopsis viridis* | 151,933 | 49.45 | 808 | 93 | 6 | 28 | 40.47 | 18,510 | NC008099 |
| *Bryopsis hypnoides* | 153,429 | 67.26 | 1496 | 69 | 5 | 37 | 33.12 | - | NC013359 |
| *Pseudendoclonium akinetum* | 195,867 | 51.90 | 968 | 105 | 6 | 31 | 31.49 | 6,039 | NC008114 |
| **Chlorophyceae** |  |  |  |  |  |  |  |  |  |
| *Chlamydomonas reinhardtii* | 203,828 | 62.11 | 1835 | 69 | 10 | 29 | 34.46 | 22,211 | NC005353 |
| *Gonium pectorale* | 222,582 | 61.36 | 2008 | 68 | 10 | 29 | 29.84 | 14,800 | NC020438 |
| *Dunaliella salina* | 269,044 | 64.93 | 2130 | 82 | 6 | 30 | 32.08 | 14,409 | NC016732 |
| *Pleodorina starrii* | 269,857 | 62.77 | 2017 | 84 | 6 | 28 | 34.95 | 16,608 | NC021109 |
| *Volvox carteri* | ~525,000 | 80.36 | 5076 | 73 | 10 | 28 | 42.41 | >25,000 | GU084820 |
| *Schizomeris leibleinii* | 182,759 | 45.66 | 1084 | 77 | 3 | 30 | 27.24 | - | NC015645 |
| *Stigeoclonium helveticum* | 223,902 | 54.45 | 1543 | 79 | 3 | 28 | 28.87 | - | NC008372 |
| *Floydiella terrestris* | 521,168 | 81.82 | 5763 | 74 | 3 | 27 | 34.53 | - | NC014346 |
| *Scenedesmus obliquus* | 161,452 | 46.02 | 965 | 77 | 6 | 30 | 26.89 | 12,022 | NC008101 |
| *Oedogonium cardiacum* | 196,547 | 39.51 | 784 | 99 | 6 | 33 | 29.52 | 35,492 | NC011031 |
| **Prasinophyceae** |  |  |  |  |  |  |  |  |  |
| *Ostreococcus tauri* | 71,666 | 35.92 | 422 | 61 | 6 | 27 | 39.89 | 6,825 | NC008289 |
| *Micromonas* sp. RCC299 | 72,585 | 45.14 | 575 | 57 | 6 | 26 | 38.85 | 4.583 | NC012575 |
| *Pycnococcus provasolii* | 80,211 | 22.71 | 268 | 68 | 2 | 30 | 39.50 | - | NC012097 |
| *Picocystis salinarum* | 81,133 | 24.0 | 243 | 80 | 6 | 35 | 37.3 | 10,364 | NC024828 |
| *Pedinomonas minor* | 98,340 | 37.59 | 451 | 82 | 6 | 36 | 34.84 | 10,639 | NC016733 |
| *Pyramimonas parkeae* | 101,605 | 30.64 | 331 | 94 | 4 | 32 | 34.70 | 13,057 | NC012099 |
| *Monomastix sp.* | 114,528 | 49.90 | 697 | 82 | 3 | 26 | 38.97 | - | NC012101 |
| *Nephroselmis olivacea* | 200,799 | 37.50 | 486 | 155 | 6 | 38 | 42.14 | 46,137 | NC000927 |

^a^Conserved genes, unique ORFs and intronic ORFs were considered as coding sequences.

^b,c^Genes present in the IR were counted twice.

**Supplementary Table S4 | Comparison of protein-coding gene contents in green algal plastid genomes**

|  | **Name** | **APRO** | **CVAR** | **CVUL** | **PKES** | **CSPA** | **PWIC** | **HSIM** | **CSPC** | **CREI** | **OTAU** | **LTER** | **PMIN** | **PAKI** | **NOLI** | **MVIR** | **CHAR** |
| --- | --- | --- | --- | --- | --- | --- | --- | --- | --- | --- | --- | --- | --- | --- | --- | --- | --- |
| **Photosynthesis** | | | | | | | | | | | | | | | | | |
|  | *psaA* | ● | ● | ● | ● | ● | ○ | ○ | ● | ● | ● | ● | ● | ● | ● | ● | ● |
|  | *psaB* | ● | ● | ● | ● | ● | ○ | ○ | ● | ● | ● | ● | ● | ● | ● | ● | ● |
|  | *psaC* | ● | ● | ● | ● | ● | ○ | ○ | ● | ● | ● | ● | ● | ● | ● | ● | ● |
|  | *psaI* | ● | ● | ●* | ● | ● | ○ | ○ | ● | ○ | ● | ● | ● | ● | ● | ● | ● |
|  | *psaJ* | ● | ● | ● | ● | ● | ○ | ○ | ● | ● | ● | ● | ● | ● | ● | ● | ● |
|  | *psaM* | ● | ● | ● | ● | ● | ○ | ○ | ● | ○ | ● | ● | ● | ●* | ○ | ●* | ●* |
|  | *psbA* | ● | ● | ● | ● | ● | ○ | ○ | ● | ● | ● | ● | ● | ● | ● | ● | ● |
|  | *psbB* | ● | ● | ● | ● | ● | ○ | ○ | ● | ● | ● | ● | ● | ● | ● | ● | ● |
|  | *psbC* | ● | ● | ● | ● | ● | ○ | ○ | ● | ● | ● | ● | ● | ● | ● | ● | ● |
|  | *psbD* | ● | ● | ● | ● | ● | ○ | ○ | ● | ● | ● | ● | ● | ● | ● | ● | ● |
|  | *psbE* | ● | ● | ● | ● | ● | ○ | ○ | ● | ● | ● | ● | ● | ● | ● | ● | ● |
|  | *psbF* | ● | ● | ● | ● | ● | ○ | ○ | ● | ● | ● | ● | ● | ● | ● | ● | ● |
|  | *psbH* | ● | ● | ● | ● | ● | ○ | ○ | ● | ● | ● | ● | ● | ● | ● | ● | ● |
|  | *psbI* | ● | ● | ● | ● | ● | ○ | ○ | ● | ● | ● | ● | ● | ● | ● | ● | ● |
|  | *psbJ* | ● | ● | ● | ● | ● | ○ | ○ | ● | ● | ● | ● | ● | ● | ● | ● | ● |
|  | *psbK* | ● | ● | ● | ● | ● | ○ | ○ | ● | ● | ● | ● | ● | ● | ● | ● | ● |
|  | *psbL* | ● | ● | ● | ● | ● | ○ | ○ | ● | ● | ● | ● | ● | ● | ● | ● | ● |
|  | *psbM* | ● | ● | ● | ● | ● | ○ | ○ | ● | ● | ○ | ● | ● | ● | ● | ●* | ● |
|  | *psbN* | ● | ● | ● | ● | ● | ○ | ○ | ● | ● | ● | ● | ● | ● | ● | ● | ● |
|  | *psbT* | ● | ● | ● | ● | ● | ○ | ○ | ● | ● | ● | ● | ● | ● | ● | ● | ● |
|  | *psbZ* | ● | ● | ● | ● | ● | ○ | ○ | ● | ● | ● | ● | ● | ● | ● | ● | ● |
|  | *petA* | ● | ● | ● | ● | ● | ○ | ○ | ● | ● | ● | ● | ● | ● | ● | ● | ● |
|  | *petB* | ● | ● | ● | ● | ● | ○ | ○ | ● | ● | ● | ● | ● | ● | ● | ● | ● |
|  | *petD* | ● | ● | ● | ● | ● | ○ | ○ | ● | ● | ○ | ● | ● | ● | ● | ● | ● |
|  | *petG* | ● | ● | ● | ● | ● | ○ | ○ | ● | ● | ● | ● | ● | ● | ● | ● | ● |
|  | *petL* | ● | ●* | ●* | ●* | ●* | ○ | ○ | ●* | ●* | ○ | ●* | ●* | ●* | ●* | ●* | ●* |
|  | *petN* | ○ | ○ | ○ | ○ | ○ | ○ | ○ | ○ | ○ | ○ | ○ | ○ | ○ | ● | ○ | ● |
|  | *atpA* | ● | ● | ● | ● | ● | ● | ○ | ● | ● | ● | ● | ● | ● | ● | ● | ● |
|  | *atpB* | ● | ● | ● | ● | ● | ● | ○ | ● | ● | ● | ● | ● | ● | ● | ● | ● |
|  | *atpE* | ● | ● | ● | ● | ● | ● | ○ | ● | ● | ● | ● | ● | ● | ● | ● | ● |
|  | *atpF* | ● | ● | ● | ● | ● | ● | ○ | ● | ● | ● | ● | ● | ● | ● | ● | ● |
|  | *atpH* | ● | ● | ● | ● | ● | ● | ○ | ● | ● | ● | ● | ● | ● | ● | ● | ● |
|  | *atpI* | ● | ● | ● | ● | ● | ● | ○ | ● | ● | ● | ● | ● | ● | ● | ● | ● |
|  | *chlB* | ● | ● | ● | ● | ● | ○ | ○ | ● | ● | ○ | ● | ○ | ○ | ● | ● | ● |
|  | *chlI* | ● | ● | ● | ● | ● | ○ | ○ | ● | ○ | ○ | ○ | ● | ● | ● | ● | ● |
|  | *chlL* | ● | ● | ● | ● | ● | ○ | ○ | ● | ● | ○ | ● | ○ | ○ | ● | ● | ● |
|  | *chlN* | ● | ● | ● | ● | ● | ○ | ○ | ● | ● | ○ | ● | ○ | ○ | ● | ● | ● |
|  | *rbcL* | ● | ● | ● | ● | ● | ○ | ○ | ● | ● | ● | ● | ● | ● | ● | ● | ● |
| **Ribosomal proteins** | | | | | | | | | | | | | | | | | |
|  | *rpl2* | ● | ● | ● | ● | ● | ● | ● | ● | ● | ● | ● | ● | ● | ● | ● | ● |
|  | *rpl5* | ● | ● | ● | ● | ● | ● | ● | ● | ● | ● | ● | ● | ● | ● | ● | ● |
|  | *rpl12* | ● | ● | ● | ● | ● | ● | ● | ● | ○ | ○ | ● | ● | ● | ● | ○ | ● |
|  | *rpl14* | ● | ● | ● | ● | ● | ● | ● | ● | ● | ● | ● | ● | ● | ● | ● | ● |
|  | *rpl16* | ● | ● | ● | ● | ● | ● | ● | ● | ● | ●* | ● | ● | ● | ● | ● | ● |
|  | *rpl19* | ● | ● | ● | ● | ● | ● | ○ | ● | ○ | ○ | ● | ● | ● | ● | ● | ● |
|  | *rpl20* | ● | ● | ● | ● | ● | ● | ● | ● | ● | ● | ● | ● | ● | ● | ● | ● |
|  | *rpl22* | ○ | ○ | ○ | ○ | ○ | ○ | ○ | ○ | ○ | ○ | ○ | ○ | ○ | ○ | ● | ● |
|  | *rpl23* | ● | ● | ● | ● | ● | ● | ○ | ● | ● | ● | ● | ● | ● | ● | ● | ● |
|  | *rpl32* | ● | ● | ● | ● | ● | ● | ● | ●* | ○ | ●* | ●* | ●* | ●* | ●* | ● | ●* |
|  | *rpl33* | ○ | ○ | ○ | ○ | ○ | ○ | ○ | ○ | ○ | ○ | ○ | ○ | ○ | ○ | ● | ● |
|  | *rpl36* | ● | ● | ● | ● | ● | ● | ● | ● | ● | ● | ● | ● | ● | ● | ● | ● |
|  | *rps2* | ● | ● | ● | ● | ● | ● | ○ | ● | ● | ● | ● | ● | ● | ● | ● | ● |
|  | *rps3* | ● | ● | ● | ● | ● | ● | ● | ● | ●* | ● | ● | ● | ● | ● | ● | ● |
|  | *rps4* | ● | ● | ● | ● | ● | ● | ● | ● | ● | ● | ● | ● | ● | ● | ● | ● |
|  | *rps7* | ● | ● | ● | ● | ● | ● | ● | ● | ● | ● | ● | ● | ● | ● | ● | ● |
|  | *rps8* | ● | ● | ● | ● | ● | ● | ● | ● | ● | ● | ● | ● | ● | ● | ● | ● |
|  | *rps9* | ● | ● | ● | ● | ● | ● | ○ | ● | ● | ● | ● | ● | ● | ● | ● | ○ |
|  | *rps11* | ● | ● | ● | ● | ● | ● | ● | ● | ● | ● | ● | ● | ● | ● | ● | ● |
|  | *rps12* | ● | ● | ● | ● | ● | ● | ● | ● | ● | ● | ● | ● | ● | ● | ● | ●* |
|  | *rps14* | ● | ● | ● | ● | ● | ● | ● | ● | ● | ● | ● | ● | ● | ● | ● | ● |
|  | *rps16* | ○ | ○ | ○ | ○ | ○ | ○ | ○ | ○ | ○ | ○ | ○ | ○ | ○ | ○ | ● | ● |
|  | *rps18* | ● | ● | ● | ● | ● | ● | ○ | ● | ● | ● | ● | ● | ● | ● | ● | ● |
|  | *rps19* | ● | ● | ● | ● | ● | ● | ●* | ● | ● | ● | ● | ● | ● | ● | ● | ● |

**Supplementary Table S4 | Comparison of protein-coding gene contents in green algal plastid genomes (Continued)**

|  | **Name** | **APRO** | **CVAR** | **CVUL** | **PKES** | **CSPA** | **PWIC** | **HSIM** | **CSPC** | **CREI** | **OTAU** | **LTER** | **PMIN** | **PAKI** | **NOLI** | **MVIR** | **CHAR** | |
| --- | --- | --- | --- | --- | --- | --- | --- | --- | --- | --- | --- | --- | --- | --- | --- | --- | --- | --- |
| **Transcription/translation** | | | | | | | | | | | | | | | | | |  |
|  | *rpoA* | ● | ● | ● | ● | ● | ● | ●* | ● | ●* | ● | ● | ● | ● | ● | ● | ● | |
|  | *rpoB* | ● | ● | ● | ● | ● | ● | ● | ● | ● | ● | ● | ● | ● | ● | ● | ● | |
|  | *rpoC1* | ● | ● | ● | ● | ● | ● | ●* | ● | ● | ●* | ●* | ● | ● | ● | ● | ● | |
|  | *rpoC2* | ● | ● | ● | ● | ● | ● | ● | ● | ●* | ● | ●* | ● | ●* | ● | ● | ● | |
|  | *infA* | ● | ● | ● | ● | ● | ● | ○ | ● | ○ | ● | ● | ○ | ○ | ● | ● | ● | |
|  | *tufA* | ● | ● | ● | ● | ● | ● | ● | ● | ● | ● | ● | ● | ● | ● | ● | ● | |
| **Division** | | | | | | | | | | | | | | | | | |  |
|  | *minD* | ● | ● | ● | ● | ● | ● | ○ | ● | ○ | ○ | ● | ● | ● | ● | ● | ○ | |
|  | *ftsH* | ● | ● | ● | ● | ● | ● | ●* | ●* | ○ | ○ | ●* | ● | ○ | ●* | ○ | ●* | |
| **Miscellaneous proteins** | | | | | | | | | | | | | | | | | |  |
|  | *accD* | ● | ● | ● | ● | ● | ● | ● | ● | ○ | ○ | ● | ● | ● | ● | ○ | ● | |
|  | *ccsA* | ● | ● | ● | ● | ● | ○ | ○ | ● | ● | ○ | ○ | ● | ● | ● | ● | ● | |
|  | *cemA* | ● | ● | ● | ● | ● | ○ | ○ | ● | ● | ○ | ● | ● | ● | ● | ● | ● | |
|  | *clpP* | ● | ● | ● | ● | ● | ● | ○ | ● | ● | ● | ● | ● | ● | ● | ● | ● | |
|  | *cysA* | ○ | ● | ● | ● | ● | ○ | ○ | ● | ○ | ○ | ● | ● | ○ | ● | ● | ○ | |
|  | *cysT* | ● | ● | ● | ● | ● | ● | ● | ● | ○ | ○ | ● | ● | ○ | ● | ● | ○ | |
|  | *ftsI* | ○ | ○ | ○ | ○ | ○ | ○ | ○ | ○ | ○ | ○ | ○ | ○ | ○ | ● | ● | ○ | |
|  | *ftsW* | ○ | ○ | ○ | ○ | ○ | ○ | ○ | ○ | ○ | ○ | ○ | ○ | ○ | ● | ● | ○ | |
|  | *odpB* | ○ | ○ | ○ | ○ | ○ | ○ | ○ | ○ | ○ | ○ | ○ | ○ | ○ | ○ | ● | ● | |
|  | *tilS/ycf62* | ● | ●* | ●* | ● | ● | ● | ● | ● | ○ | ○ | ● | ○ | ○ | ● | ● | ● | |
|  | I-CvuI | ○ | ○ | ● | ○ | ○ | ○ | ○ | ○ | ○ | ○ | ○ | ○ | ● | ○ | ○ | ○ | |
| **NADH oxidoreductase** | | | | | | | | | | | | | | | | | |  |
|  | *ndhA* | ○ | ○ | ○ | ○ | ○ | ○ | ○ | ○ | ○ | ○ | ○ | ○ | ○ | ●* | ● | ● | |
|  | *ndhB* | ○ | ○ | ○ | ○ | ○ | ○ | ○ | ○ | ○ | ○ | ○ | ○ | ○ | ● | ● | ● | |
|  | *ndhD* | ○ | ○ | ○ | ○ | ○ | ○ | ○ | ○ | ○ | ○ | ○ | ○ | ○ | ● | ● | ● | |
|  | *ndhE* | ○ | ○ | ○ | ○ | ○ | ○ | ○ | ○ | ○ | ○ | ○ | ○ | ○ | ●* | ● | ● | |
|  | *ndhF* | ○ | ○ | ○ | ○ | ○ | ○ | ○ | ○ | ○ | ○ | ○ | ○ | ○ | ● | ● | ● | |
|  | *ndhG* | ○ | ○ | ○ | ○ | ○ | ○ | ○ | ○ | ○ | ○ | ○ | ○ | ○ | ● | ● | ● | |
|  | *ndhH* | ○ | ○ | ○ | ○ | ○ | ○ | ○ | ○ | ○ | ○ | ○ | ○ | ○ | ● | ● | ● | |
|  | *ndhI* | ○ | ○ | ○ | ○ | ○ | ○ | ○ | ○ | ○ | ○ | ○ | ○ | ○ | ● | ● | ● | |
|  | *ndhJ* | ○ | ○ | ○ | ○ | ○ | ○ | ○ | ○ | ○ | ○ | ○ | ○ | ○ | ○ | ● | ● | |
|  | *ndhK* | ○ | ○ | ○ | ○ | ○ | ○ | ○ | ○ | ○ | ○ | ○ | ○ | ○ | ● | ● | ● | |
| **Conserved proteins** | | | | | | | | | | | | | | | | | |  |
|  | *ycf1* | ● | ● | ● | ● | ● | ● | ●* | ● | ○ | ○ | ● | ● | ● | ●* | ●* | ●* | |
|  | *ycf3* | ● | ● | ● | ● | ● | ○ | ○ | ● | ● | ● | ● | ● | ● | ● | ● | ● | |
|  | *ycf4* | ● | ● | ● | ● | ● | ○ | ○ | ● | ● | ○ | ● | ● | ● | ● | ● | ● | |
|  | *ycf12* | ● | ● | ● | ● | ● | ○ | ○ | ● | ●* | ●* | ○ | ● | ● | ●* | ●* | ●* | |
|  | *ycf20* | ○ | ● | ● | ●* | ● | ○ | ○ | ● | ○ | ○ | ● | ● | ●* | ○ | ● | ● | |
|  | *ycf47* | ○ | ● | ● | ● | ● | ○ | ○ | ● | ○ | ○ | ○ | ● | ○ | ○ | ○ | ○ | |
|  | *ycf66* | ○ | ○ | ○ | ○ | ○ | ○ | ○ | ○ | ○ | ○ | ○ | ○ | ○ | ○ | ● | ● | |

● indicate presence of genes which protein sequence can alignment with E-value >= 1e-7 and alignment region in both genes >=1/4; ○ indicate absence of genes;

* indicate E-value < 1e-7 or alignment region in both genes <1/4 compared to APRO.

The genes only contained in one species were not shown.

Abbreviations for species: APRO: *Auxenchlorella protothecoides*; CVAR: *Chlorella variabilis*; CVUL: *Chlorella vulgaris*; PKES: *Parachlorella kessleri*; CSPA: *Chlorella* sp. ArM0029B; PWIC: *Prototheca wickerhamii*; HSIM: Helicosporidium sp; CSPC: *Coccomyxa* sp C-169; CREI: *Chlamydomonas reinhardtii*; NOLI: *Nephroselmis olivacea*; OTAU: *Ostreococcus tauri*; LTER: *Leptosira terrestris*; PMIN: *Pedinomonas minor*; PAKI : *Pseudendoclonium akinetum*; MVIR: *Mesostigma viride*; CHAR: *Chara vulgaris*; CSPA: *Chlorella sp. ArM0029B*

**Supplementary Table S5 | Comparison of RNA gene contents in green algal plastid genomes**

|  | **Gene name** | **APRO** | **CVAR** | **CVUL** | **PKES** | **CSPA** | **PWIC** | **HSIM** | **CSPC** | **CREI** | **OTAU** | **LTER** | **MVIR** | **PMIN** | **NOLI** | **PAKI** | **CHAR** | |
| --- | --- | --- | --- | --- | --- | --- | --- | --- | --- | --- | --- | --- | --- | --- | --- | --- | --- | --- |
| **Ribosomal RNAs** | | | | | | | | | | | | | | | | | | |
|  | *rrn5* | ● | ● | ● | ● | ● | ● | ● | ● | ● | ● | ● | ● | ● | ● | ● | ● | |
|  | *rnl* | ● | ● | ● | ● | ● | ● | ● | ● | ● | ● | ● | ● | ● | ● | ● | ● | |
|  | *rns* | ● | ● | ● | ● | ● | ● | ● | ● | ● | ● | ● | ● | ● | ● | ● | ● | |
|  | *rrn3* | ○ | ○ | ○ | ○ | ○ | ○ | ○ | ○ | ● | ○ | ○ | ○ | ○ | ○ | ○ | ○ | |
|  | *rrn7* | ○ | ○ | ○ | ○ | ○ | ○ | ○ | ○ | ● | ○ | ○ | ○ | ○ | ○ | ○ | ○ | |
| **Transfer RNAs** | | | | | | | | | | | | | | | | | | |
|  | *trnT* | ● | ● | ● | ● | ● | ● | ● | ● | ● | ● | ● | ● | ● | ● | ● | ● | |
|  | *trnR* | ● | ● | ● | ● | ● | ● | ● | ● | ● | ● | ● | ● | ● | ● | ● | ● | |
|  | *trnS* | ● | ● | ● | ● | ● | ● | ● | ● | ● | ● | ● | ● | ● | ● | ● | ● | |
|  | *trnP* | ● | ● | ● | ● | ● | ● | ● | ● | ● | ● | ● | ● | ● | ● | ● | ● | |
|  | *trnW* | ● | ● | ● | ● | ● | ● | ● | ● | ● | ● | ● | ● | ● | ● | ● | ● | |
|  | *trnE* | ● | ● | ● | ● | ● | ● | ● | ● | ● | ● | ● | ● | ● | ● | ● | ● | |
|  | *trnM* | ● | ● | ● | ● | ● | ● | ● | ● | ● | ● | ● | ● | ● | ● | ● | ● | |
|  | *trnG* | ● | ● | ● | ● | ● | ● | ● | ● | ● | ● | ● | ● | ● | ● | ● | ● | |
|  | *trnY* | ● | ● | ● | ● | ● | ● | ● | ● | ● | ● | ● | ● | ● | ● | ● | ● | |
|  | *trnA* | ● | ● | ● | ● | ● | ● | ● | ● | ● | ● | ● | ● | ● | ● | ● | ● | |
|  | *trnI* | ● | ● | ● | ● | ● | ● | ● | ● | ● | ● | ● | ● | ● | ● | ● | ● | |
|  | *trnN* | ● | ● | ● | ● | ● | ● | ● | ● | ● | ● | ● | ● | ● | ● | ● | ● | |
|  | *trnL* | ● | ● | ● | ● | ● | ● | ● | ● | ● | ● | ● | ● | ● | ● | ● | ● | |
|  | *trnQ* | ● | ● | ● | ● | ● | ● | ● | ● | ● | ● | ● | ● | ● | ● | ● | ● | |
|  | *trnF* | ● | ● | ● | ● | ● | ● | ● | ● | ● | ● | ● | ● | ● | ● | ● | ● | |
|  | *trnK* | ● | ● | ● | ● | ● | ● | ● | ● | ● | ● | ● | ● | ● | ● | ● | ● | |
|  | *trnH* | ● | ● | ● | ● | ● | ● | ● | ● | ● | ● | ● | ● | ● | ● | ● | ● | |
|  | *trnV* | ● | ● | ● | ● | ● | ● | ● | ● | ● | ● | ● | ● | ● | ● | ● | ● | |
|  | *trnC* | ● | ● | ● | ● | ● | ● | ● | ● | ● | ● | ● | ● | ● | ● | ● | ● | |
|  | *trnD* | ● | ● | ● | ● | ● | ● | ● | ● | ● | ● | ● | ● | ● | ● | ● | ● |  |

● indicate presence of genes and ○ indicate absence of genes as in Supplemental Table 4.

Abbreviations for species: APRO: *Auxenchlorella protothecoides*; CVAR: *Chlorella variabilis*; CVUL: *Chlorella vulgaris*; PKES: *Parachlorella kessleri*; CSPA: *Chlorella* sp. *ArM0029B; PWIC: Prototheca wickerhamii; HSIM: Helicosporidium sp; CSPC: Coccomyxa sp. C-169; CREI: Chlamydomonas reinhardtii; NOLI: Nephroselmis olivacea; OTAU: Ostreococcus tauri; LTER: Leptosira terrestris; PMIN: Pedinomonas minor; PAKI: Pseudendoclonium akinetum; MVIR: Mesostigma viride; CHAR: Chara vulgaris;*

**Supplementary Table S6 | Genes lost from *P. wickerhamii* plastid genome compared to *A. protothecoides***

| **Breakpoint** | **Genes** | **Length(bp)** |
| --- | --- | --- |
| BP1 | *ycf3*-*pbsI* | 786 |
| BP2 | *chlI*-*petA*-*petL*-*petG*-*psbD*-*psbC* | 5286 |
| BP3 | *cemA*-*ycf4* | 1339 |
| *BP4* | *psaJ* | 188 |
| BP5 | *psbM* | 199 |
| BP6 | *psbH*-*psbN*-*psbT*-*psbB* | 2574 |
| BP7 | *ccsA*-*chlL*-*chlN* | 3637 |
| BP8 | *psaC* | 386 |
| BP9 | *trnL*-*trnS* | 128 |
| BP10 | *psaB*-*psaA*-*chlB*-*psbK*-*ycf12*-*psaM* | 7504 |
| BP11 | *psbZ* | 217 |
| BP12 | *psbE*-*psbF*-*psbL*-*psbJ* | 761 |
| BP13 | *trnT(GGU)* | 236 |
| BP14 | *psaI* | 135 |
| BP15 | *petD*-*petB* | 1297 |
| BP16 | *psbA* | 1010 |
| BP17 | *rbcL* | 1638 |
